# Smell as a Disease Marker in Multiple Sclerosis

**DOI:** 10.3390/jcm11175215

**Published:** 2022-09-03

**Authors:** Athanasia Printza, Marina Boziki, Constantinos Valsamidis, Christos Bakirtzis, Jannis Constantinidis, Nikolaos Grigoriadis, Stefanos Triaridis

**Affiliations:** 1 1st Otolaryngology Department, School of Medicine, Faculty of Health Sciences, Aristotle University of Thessaloniki, AHEPA Hospital, 54124 Thessaloniki, Greece; 2 2nd Neurology Department, School of Medicine, Faculty of Health Sciences, Aristotle University of Thessaloniki, AHEPA Hospital, 54124 Thessaloniki, Greece

**Keywords:** smell, olfaction, multiple sclerosis, olfactory threshold, identification, nasal symptoms, quality of life, cognitive function, patient-reported-outcome measures, disease marker

## Abstract

Existing data suggest that people with multiple sclerosis (pwMS) are at an elevated risk for experiencing olfactory impairment. We investigated if smell dysfunction can be used as an MS disease marker. This is a cross-sectional, case–control study. All data were collected prospectively from 171 participants, 115 pwMS and 56 controls (age and sex stratified and matched to the patients), who reported smell, taste, and nasal breathing, and completed the Greek-validated questionnaires for nasal obstruction (NOSE), nasal-symptoms QoL (SNOT-22), and olfaction-associated QoL (QOD). The smell was assessed with the “Sniffin’ sticks” (odor threshold (OT), discrimination (OD), identification (OI) test, and total TDI). We recorded the pwMS disease characteristics (Expanded Disability Status Scale-EDSS, the disease type and duration), cognitive function, emotional status, fatigue, and impact of MS in everyday activities. A TDI < 30.75 (hyposmia) was detected in 30.8% of the patients. The patients’ OD and TDI scores were significantly lower than the controls’ (*p* = 0.005, and 0.015, respectively). The hyposmia correlated with disease severity and duration. The EDSS score correlated negatively with OD (r = −0.299, *p* = 0.001) and TDI (r = −0.242, *p* = 0.01). The disease duration correlated negatively with OD (r = −0.305, *p* = 0.001, OI (r = −0.253, *p* = 0.008) and TDI (r = −0.3, *p* = 0.001). The information processing speed (SDMT) correlated with OD, OT, and TDI (r = 0.302, *p* = 0.002; r = 0.242, *p* = 0.016; r = 0.326, *p* = 0.001). The olfactory function is changing in MS in accordance with disease progression.

## 1. Introduction

Smell disorders have been increasingly recognized in different neuroinflammatory and neurodegenerative diseases. In multiple sclerosis (MS), studies have reported prevalence rates of olfactory dysfunction ranging from 11% to 93% [1]. Existing evidence suggests that smell impairment might be an under-recognized problem, but there are significant inconsistencies in the literature regarding the true extent of the issue [1,2,3,4]. These discrepancies may stem in part from the testing modalities that were used to measure the olfaction, small sample sizes, and the varying baseline disease characteristics of the patient populations studied. Most studies report Threshold–Discrimination–Identification scores (TDI scores), measuring the olfactory function with the “Sniffin’ Sticks” [4,5], some used the University of Pennsylvania Smell Identification Test (UPSIT) [6,7], and a number of studies used different tests measuring odor identification, threshold, or combinations with extensive or shorter screening tests [1,7]. Sample sizes in studies range from 14 to 153 patients, and the patient populations studied have varying disease characteristics. Only a few studies examined cognitive [4,7] and emotional [1] status, and there is lack of information regarding confounding factors related to nasal function. Our study examined if smell dysfunction in multiple sclerosis can be used as a disease marker. We examined correlations of the hyposmia to the MS type, Expanded Disability Status Scale (EDSS), disease duration, cognitive and emotional status, and evaluated the nasal-function-related confounding factors as well as the nasal-symptoms/olfaction-associated quality of life (QoL). 

## 2. Materials and Methods

This is a cross-sectional case–control study. The study was approved by the Institutional Review Board and was carried out according to the Declaration of Helsinki. All the participants provided written informed consent prior to enrollment in the study. All data were collected prospectively from 171 participants, 115 people with MS (pwMS) and 56 controls (age and sex stratified and matched to the patients). All participants were recruited on a consecutive basis, as the study was conducted in the frame of real-world clinical practice.

Inclusion criteria for the pwMS subgroup were a diagnosis of MS according to the revised 2017 McDonald Criteria, and adult age. The patients were consecutively recruited from the Multiple Sclerosis Center of AHEPA University Hospital during their regular visits. The healthy participants were community-dwelling adult volunteers with no history of smell impairment. Exclusion criteria were a diagnosis of cognitive impairment and a history of diseases, surgery, or toxic exposure which can impair smell. Moreover, patients upon disease relapse and participants with an active infection, clinically or laboratory confirmed, were excluded from the study. All patients were in disease remission, taking into consideration the absence of clinical relapse as well as absence of MRI markers of disease activity. No additional measurements of white matter lesion and/or volumetric analysis were conducted. 

The participants were asked to report smell disorders, taste disorders, nasal breathing difficulties, and nasal discharge by means of self-assessment at a visual analogue scale (VAS), and completed the Greek validated versions of questionnaires for nasal obstruction (Nasal Obstruction Symptom Evaluation: NOSE) [8,9], for nasal-symptoms-associated QoL (SinoNasal Outcome Test-22: SNOT-22) [10,11], and for olfaction-associated QoL (Questionnaire of Olfactory Deficits: QOD) [12,13]. The olfactory function of all participants was quantitatively assessed using the “Sniffin’ sticks” test (Burghart, Wedel, Germany), that includes specific tests for odor threshold (OT), discrimination (OD), and identification (OI) assessment [5,14,15]. The scores were summed up to a “Threshold Discrimination Identification (TDI) score”. TDI score ranges from 0 to 48. Values of 16 or less represent anosmia, values between 16 and 30.5 represent hyposmia, and values over 30.75 represent normosmia [16].

An experienced Expanded Disability Status Scale (EDSS)-certified neurologist performed the EDSS evaluation of the pwMS. The patients’ cognitive function (information processing speed, verbal memory, visuospatial memory), depressive symptoms, fatigue, and impact of MS in everyday activities were recorded by the treating team of neurologists. All pwMS underwent cognitive screening, using the Greek version of the BICAMS battery [17,18,19], which consists of three tests: (a) The Symbol Digit Modalities Test (SDMT) [20,21] as a measure of information processing speed, (b) The Greek Verbal Learning Test (GVLT) [22] as a measure of verbal memory, and (c) The Brief Visuospatial Memory Test–Revised (BVMT-R) [23] as a measure of visuospatial memory. Additionally, the pwMS were administered the Modified Fatigue Impact Scale (MFIS) [24] as a measure of self-reported fatigue, the Beck Depression Inventory-Fast Screen (BDI-FS) [25] as a measure of mood, and the Multiple Sclerosis Impact Scale-29 (MSIS-29) [26] in order to quantify the impact of MS symptoms on everyday life activities. The MFIS was used for a more comprehensive assessment of fatigue in MS [27]. A value greater than 38 is indicative of fatigue. The BDI-FS questionnaire has been previously validated for use in multiple sclerosis [28]. It consists of 7 items with a maximum score of 21. Scores of 0–3 indicate minimal depression and a diagnostic cut-off value is ≥4.

Data regarding the demographic characteristics of all the participants, smoking, and the clinical characteristics of the MS patients were recorded, including the type of MS, EDSS score, and disease duration.

The pwMS olfactory function was measured and compared to controls. The incidence of hyposmia and impairment of odor threshold (OT), discrimination (OD), and identification (OI) were measured. The confounding factors related to nasal function were evaluated. The pwMS group was further divided into hyposmic and normosmic subgroups (TDI values over 30.75). The nasal-symptoms-associated QoL and olfaction-associated QoL were evaluated. The olfactory dysfunction was correlated to nasal symptoms, MS type, EDSS, disease duration, cognitive function, and emotional status.

### Statistical Analysis

For the comparison of self-reported smell disorders, taste disorders, nasal breathing difficulties, and nasal discharge between pwMS and controls, Pearson’s chi square test was implemented, as the self-reported results were handled as categorical variables. For the comparison of gender distribution between cases and controls, as well as across MS type, Pearson’s chi square test was implemented. For the comparison of age, SNOT22, NOSE, identification score, discrimination score, QoD, threshold score, and TDI between pwMS and controls, either independent samples using the Student’s T-test or Mann–Whitney U Test were conducted following Kolmogorov–Smirnov Normality tests. For the comparison of anosmic/hyposmic vs. normosmic, as well as anosmic vs. hyposmic vs. normosmic distribution of individuals between cases and controls, Pearson’s chi square test was implemented. This was similarly conducted for the comparison of anosmic/hyposmic vs. normosmic as well as anosmic vs. hyposmic vs. normosmic distribution of individuals among MS type. Spearman’s correlations were conducted only for pwMS in order to investigate potential correlations between age, EDSS score, disease duration, cognition measurements, and olfaction scores. Binary Logistic Regression analysis was conducted in order to explore possible effect of factors on the olfactory outcome: the TDI-based patient distribution into anosmic/hyposmic vs. normosmic was set as a dependent variable. Two models were implemented: one with age, gender, disease duration, MS type, EDSS score, smoking status, SDMT, GVLT, BVMT-R, MFIS, BDI, and MSIS as independent variables, and a second model with age, gender, disease duration, MS type, EDSS score, and smoking status as independent variables. Probabilities were calculated for each model and the individual probability value was plotted against the binary anosmic/hyposmic vs. normosmic status in order to obtain ROC curves. Statistical significance was set at α = 0.05 for all tests.

## 3. Results

The study cohort comprised of 171 participants, 115 pwMS and 56 healthy controls, 57 male and 114 female participants (40 male and 75 female pwMS, and 17 male and 39 female controls). Female participants represented 65.2% of the pwMS and 69.6% of healthy controls. The patients’ ages ranged from 16 to 68 (mean 38.69 ± 12.19), and the controls’ ages ranged from 19 to 61 (mean 37.29 ± 12.01).

There was no significant difference between the two groups concerning gender and age (*p* > 0.05). The patients’ group was further divided to hyposmic and normosmic subgroups (TDI values over 30.75). The average time from the onset of MS symptoms was 10.08 years (SD 8.47 years). The average time from diagnosis was 7.74 years (SD 7.8 years). The mean EDSS was 3.7 (SD 2.02). The demographic and clinical data of the participants are presented in Table 1.The MS group patients were diagnosed with Relapsing Remitting MS-RRMS (83 patients, 72.17%), Primary Progressive MS-PPMS (16 patients, 13.9%), and Secondary Progressive MS-SPMS (16 patients, 13.9%).

Regarding the self-reported nasal function, as it was recorded on a 0–4 symptom scale for olfactory dysfunction, taste disorders, nasal breathing difficulty, and nasal discharge, both groups of participants reported minimal symptoms: 92.7% of the controls and 93.9% of the patients reported minimal or no olfactory dysfunction (87.27% and 86.9%, respectively, rated it as 0), 96.4% of the controls and 93.04% of the patients reported minimal or no taste disorder (92.86% and 87.8%, respectively, rated it as 0), 92.86% of the controls and 88.7% of the patients reported minimal or no nasal discharge, and 80.36% of the controls and 96.56% of the patients reported minimal or no nasal breathing difficulties. The two groups were only found to marginally differ in the self-reported nasal breathing difficulties (Pearson’s chi square = 7.794, *p* = 0.05).

Compared to age and sex stratified normative data (A. Oleszkiewicz et al., updated Sniffin’ Sticks normative data, 2019) 18.8% of the patients were hyposmic. A TDI < 30.75 (general definition of hyposmia) was detected in 30.8% of the patients (26.5% of the patients with RRMS, 37.5% of those with PPMS, and 43.75% of those with SPMS).

We compared the patients’ olfactory ability to that of the controls. The patients’ OD and TDI scores were significantly lower than the controls’. The olfactory status at enrollment is presented in Table 2.

We evaluated the nasal-function-related confounding factors, and the nasal-symptoms/olfaction-associated QoL. The mean NOSE for the pwMS group was 13.25 (SD 17.81) and for the controls 8.96 (SD 12.63), not significantly different (Mann–Whitney U test, *p* = 0.532). This finding indicated that the two groups did not differ in nasal obstruction, which can be related to reduced olfactory ability. The mean SNOT-22 (nasal-symptoms-associated QoL) for the pwMS group was 21 (SD 17.05), significantly worse (Mann–Whitney U test, *p* < 0.001) than the controls’ mean, 10.36 (SD 12.76). Additional exploratory analysis of the SNOT-22 indicated that the two groups differed significantly in their responses about fatigue and sleep disorders. The QoD (olfaction-associated QoL) mean scores for the patients and controls were 17.49 (SD 9.12) and 17.79 (SD 9.13), respectively, which were found not to differ significantly (Mann–Whitney U test, *p* = 0.967).

The mean TDI score of pwMS with a SDMT < 45 (39.64% of pwMS) was 31.45 (SD 4.72) compared to 34.94 (SD 4.08) in pwMS with a SDMT ≥ 45. The mean TDI score of pwMS with a BDI-FS score 0–3 (45.95% of pwMS) was 33.73 (SD 4.57) compared to 32.58 (SD 4.43) in pwMS with a BDI-FS score ≥ 4.

We stratified the pwMS group into anosmic/hyposmic and normosmic. We examined correlations of the hyposmia to the MS type, EDSS scores, disease duration, and cognitive and emotional status. The distribution of MS type did not differ between anosmic/hyposmic and normosmic patients (Pearson’s chi square = 2.322, *p* = 0.313).

Regarding hyposmia and MS disease characteristics, hyposmia correlated with increased disease severity (EDSS) and increased disease duration. A lower EDSS score was correlated with increased OD (r = −0.299, *p* = 0.001) and TDI (r = −0.242, *p* = 0.01) scores. Increased disease duration correlated with reduced OD (r = −0.305, *p* = 0.001, OI (r = −0.253, *p* = 0.008) and TDI (r = −0.3, *p* = 0.001) scores. Based on the observation that hyposmia correlated with increased disease severity (EDSS) and increased disease duration, we then conducted a stratified analysis by selecting MS patients with disease duration equal to or less than 5 and 3 years, and compared the mean TDI scores between selected MS patient groups and controls, respectively. Notably, upon these comparisons, the mean TDI scores did not differ between MS and controls (34.35 ± 0.7 vs. 34.92 ± 0.57, *p* = 0.521 and 35.06 ± 0.79 vs. 34.92 ± 0.57, *p* = 0.886 for MS patients with disease duration equal to or less than 5 years vs. controls and for MS patients with disease duration equal to or less than 3 years vs. controls, respectively). The lack of statistical difference upon these comparisons may be attributed to the reduction in the MS group sample size, which is N = 46 and N = 32 for MS patients with disease duration equal to or less than 5 and 3 years, respectively, whereas the total patient number in the study is N = 115.

The information processing speed (SDMT) of pwMS was correlated with higher OD, OT, and TDI (r = 0.302, *p* = 0.002; r = 0.242, *p* = 0.016; r = 0.326, *p* = 0.001) scores. The visuospatial memory (BVMT-R) was correlated with higher TDI (r = 0.227, *p* = 0.027) score. The fatigue correlated with lower OT (Spearman’s rho = −0.221, *p* = 0.028), and exhibited a tendency to correlate with lower TDI (Spearman’s rho = −0.189, *p* = 0.061) score.

**Figure 1:**
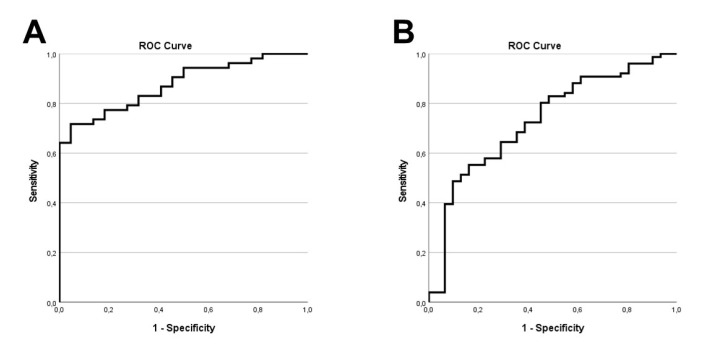
ROC curves of two probability prediction models of the olfactory outcome. Probabilities were calculated based on Binary Logistic Regression models. (**A**) age, gender, disease duration, Multiple Sclerosis disease type, EDSS score, smoking status, SDMT, GVLT, BVMT-R, MFIS, BDI, and MSIS were set as independent variables. (**B**) Age, gender, disease duration, MS type, EDSS score, and smoking status were set as independent variables. EDSS: Expanded Disability Status Scale; SDMT: Symbol Digit Modalities Test; GVLT: Greek Verbal Learning Test; BVMT-R: Brief Visuospatial Memory Test–Revised; MFIS: Modified Fatigue Impact Scale; BDI-FS: Beck Depression Inventory-Fast Screen; MSIS: Multiple Sclerosis Impact Scale.

Binary Logistic Regression model was able to predict the olfactory outcome, namely, the TDI-based patient distribution into anosmic/hyposmic vs. normosmic when age, gender, disease duration, MS type, EDSS score, smoking status, SDMT, GVLT, BVMT-R, MFIS, BDI, and MSIS were set as independent variables (R Square = 0.443, *p* = 0.009). Notably, when age, gender, disease duration, MS type, EDSS score and smoking status were set as independent variables, the model was not able to predict the olfactory outcome (R Square = 0.165, *p* = 0.069), thus underlining the importance of cognition measurements in contributing to the predictive model of the olfactory outcome. For the two models, Area Under the Curve (AUC) was 0.876 and 0.731, respectively (Figure 1).

## 4. Discussion

Existing data suggest that pwMS are at an elevated risk for experiencing olfactory impairment [2,29]. It is crucial to evaluate olfactory dysfunction as it affects the quality of life [2,30]; smell is related to a person’s physical, behavioral, emotional, and cognitive state, and also because there is growing evidence that the hyposmia in pwMS can be used as a potential disease marker [2,29].

Our study evaluated a large cohort of pwMS presenting a sex, age, and MS types distribution representative of the general MS patients’ population. Their olfactory ability was compared with recently published normative data and with a control group. A TDI < 30.75 (defining hyposmia) was detected in 30.8% of the pwMS (26.5% of the patients with RRMS, 37.5% of those with PPMS, and 43.75% of those with SPMS). Our findings are corroborated by a recently published systematic review and meta-analysis of 1099 MS cases which found that the pooled prevalence of olfactory dysfunction was 27.2% (95% CI: 19.7%, 35.4%) [29]. Compared to age- and sex-stratified updated Sniffin’ Sticks normative data, 18.8% of the patients were found to be hyposmic (scores lower than the 10th percentile [16]. Interestingly, 86.9% of the pwMS reported no olfactory dysfunction (they rated it as 0) and 93.9% reported minimal or no dysfunction. Bsteh et al. also reported that although the pwMS had significantly impaired olfactory ability, only 4.9% of those with RRMS and 3.5% of those with progressive MS noticed or described an impairment of olfactory function [2].

The discrepancies between patient-reported olfactory ability and psychophysical results have been extensively described, and are even more important for pwMS since this is a multifaceted condition possibly affecting the person’s cognition, mood, and typically inducing fatigue [2,27]. These factors further complicate the valid evaluation of olfaction and render necessary the psychophysical testing. Our regression analysis demonstrated that cognition measurements are contributing to the predictive model of the olfactory outcome.

Compared to our age- and sex-matched controls, the patients’ OD, and TDI scores were significantly lower. Mirmosayyeb et al. reported lower TDI, OD, OI, and OT patients’ scores compared to controls [29]. In accordance with our findings, Bsteh et al. published data suggesting that the odor threshold was markedly impaired in patients with relapse activity within 12 months, and recovered in the absence of relapse [2]. Odor threshold scores strongly fluctuated within subjects at repeated measurements [2]. Threshold impairment was found to be transient and predictive of inflammatory disease activity, while odor discrimination was associated with disability progression. Furthermore, while impairment of discrimination and identification were found to be associated with gray matter atrophy in brain regions related to olfactory function, threshold impairment was not associated [31].

Regarding the olfactory dysfunction and MS disease characteristics, the hyposmia (TDI) and the OD were found to correlate with the disability severity (EDSS) and the disease duration. Greater disability and longer disease duration correlated with worse olfactory ability (total score) and odor discrimination. A recent meta-analysis found that the pooled prevalence of olfactory dysfunction was higher in studies with a mean EDSS higher than three compared to those with EDSS lower than three (32.6% vs. 15.9%, *p* = 0.048) [29]. In a prospective 3-year longitudinal study on 151 pwMS, odor discrimination strongly associated with EDSS progression [2].

Our study evaluated the nasal confounding factors via self-reporting of symptoms and validated questionnaires. Regarding the self-reported nasal function, both groups of participants, pwMS and controls, reported minimal symptoms. The two groups did not differ in nasal obstruction, which can be related to reduced olfactory ability, as demonstrated by the comparison of the NOSE scores. The significant differences in SNOT-22 mean values between pwMS and controls could be taken falsely as an indication of different nasal functions contributing to the olfactory ability differences [32], if exploratory analysis of the items had not been undertaken, pointing to fatigue and sleep disorders as the symptoms responsible for higher SNOT-22 mean values in pwMS. This is a characteristic example of a rigorously developed and validated PROM [10,11,32] that can have a varying degree of validity depending on its use in a broad range of contexts, populations, and purposes [33,34].

The SDMT, a well-established and sensitive screening test for cognitive dysfunction, has been reported to correlate with the disease severity, brain atrophy, and quality of life in MS [35]. The SDMT correlated with the olfactory scores significantly and contributed to the predictive model of the olfactory ability. Smell appears to be a disease marker in MS and PROMs and olfactory ability tests may be introduced into core outcome sets used in clinical trials in MS [33].

Among the strengths of our study are the large sample of pwMS of all types, the comparison of olfaction and nasal function with a matched control group, and the comprehensive use of measures of nasal function, QoL, and neurocognitive and emotional function. A limitation of our study is that we did not analyze imaging studies, relapse history, infection history, or the effect of treatments on the olfactory ability. Notably, olfactory function has been recently associated with measurements of disability progression and the degree of brain atrophy in the MRI [31]. With respect to infectious status, all patients were evaluated in the absence of active infection, either clinically or laboratory confirmed. However, history of past infections was not evaluated. Viral infections have been advocated to be implicated in neuroinflammation and neurodegeneration, and the olfactory bulb has been suggested as a potential route of entry for infectious agents into the CNS [36], a possibility further highlighted in the era of the COVID-19 pandemic [37]. With respect to this, the addition of MRI measurements and the infection history in the overall patient evaluation is expected to be of increased value for future studies. Moreover, in the setting of the present study, a discrepancy in the sample size between PwMS and controls is evident. This fact is in accordance with the inherent limitations linked with control subjects’ recruitment for clinical studies, as all participants were recruited on a consecutive basis. Special effort was made to include matching populations in terms of gender and age, in order to yield comparable results. Notably, propensity score matching is a promising analysis method to control for confounding factors and discrepancies in the baseline characteristics between two groups under study. Although this method is increasingly applied in real-world studies, especially those including interventions, in order to correct for the absence of randomization [38] in the present case–control study, we did not conduct a propensity score matching analysis. This is a limitation in the analysis, as the inclusion of a propensity score algorithm may facilitate the reduction in selection bias in case–control studies [39]. However, as in the setting of the present study the recruitment of participants in a consecutive manner yielded groups with a discrepancy in sample size, the additional inclusion of a propensity score algorithm is expected to further reduce participants’ numbers. Larger participants’ groups which are more balanced in terms of sample size would be necessary in order to proceed with such analysis.

## 5. Conclusions

The results of this study show that the prevalence of olfactory dysfunction in pwMS is significantly higher than in the general population. Olfactory dysfunction might be a useful and easily obtainable parameter to monitor patients with regard to inflammation and neurodegeneration in MS. The olfactory function is changing in MS in accordance with disease progression and can be used as a disease marker.

## Figures and Tables

**Table 1:** Demographic and clinical data of the participants.

	MS Patients	Controls	*p*
**age**	38.69 ± 12.19	37.29 ± 12.01	0.527 *
**disease duration (years)**	10.08 ± 8.47	N/A	N/A
**disease duration since diagnosis (years)**	7.74 ± 7.80	N/A	N/A
**EDSS**	3.7 ± 2.02	N/A	N/A
**SDMT**	45.07 ± 14.24	N/A	N/A
**GVLT**	55.41 ± 11.9	N/A	N/A
**BVMT-R**	22.21 ± 8.76	N/A	N/A
**MFIS**	31.06 ± 21.76	N/A	N/A
**BDI-FS**	3.85 ± 3.78	N/A	N/A
**MSIS**	61.25 ± 22.64	N/A	N/A
**SNOT-22**	21 ± 17.05	10.36 ± 12.76	<0.001 *
**NOSE**	13.25 ± 17.81	8.96 ± 12.63	0.532 *
**QoD**	17.49 ± 9.12	17.79 ± 9.13	0.967 *

* Mann–Whitney U test; EDSS: Expanded Disability Status Scale; SDMT: Symbol Digit Modalities Test; GVLT: Greek Verbal Learning Test; BVMT-R: Brief Visuospatial Memory Test–Revised; MFIS: Modified Fatigue Impact Scale; BDI-FS: Beck Depression Inventory-Fast Screen; MSIS: Multiple Sclerosis Impact Scale; N/A: non-applicable.

**Table 2:** The olfactory status of the participants.

	MS Patients	Controls	*p*
**OD**	12.19 ± 0.23	13.3 ± 0.3	0.005 *
**OI**	14.08 ± 0.16	14.66 ± 0.15	0.06 *
**OT**	6.72 ± 0.26	6.96 ± 0.35	0.581 **
**TDI**	32.98 ± 0.48	34.92 ± 0.57	0.015 **

* Mann–Whitney U test; ** *t*–test; mean comparison test selected following normality test. OD: odor discrimination; OI: odor identification; OT: odor threshold; TDI: Threshold Discrimination Identification.

## Data Availability

Data is available from the authors at request.

## References

[1] Lucassen E.B., Turel A., Knehans A., Huang X., Eslinger P. (2016). Olfactory dysfunction in Multiple Sclerosis: A scoping review of the literature. Mult. Scler. Relat. Disord..

[2] Bsteh G., Hegen H., Ladstätter F., Berek K., Amprosi M., Wurth S., Auer M., Pauli F.D., Deisenhammer F., Reindl M. (2019). Change of olfactory function as a marker of inflammatory activity and disability progression in MS. Mult. Scler..

[3] Doty R., Li C., Mannon L., Yousem D. (1997). Olfactory dysfunction in multiple sclerosis. N. Engl. J. Med..

[4] Goektas O., Schmidt F., Bohner G., Erb K., Ludemann L., Dahlslett B., Harms L., Fleiner F. (2011). Olfactory bulb volume and olfactory function in patients with multiple sclerosis. Rhinology.

[5] Hummel T., Sekinger B., Wolf S., Pauli E., Kobal G. (1997). Sniffin’ Sticks: Olfactory performance assessed by the combined testing of odour identification, odour discrimination and olfactory threshold. Chem. Senses.

[6] Hawkes C., Shephard B., Kobal G. (1997). Assessment of olfaction in multiple sclerosis: Evidence of dysfunction by olfactory evoked response and identification tests. J. Neurol. Neurosurg. Psychiatry.

[7] Silva A., Santos E., Moreira I., Bettencourt A., Coutinho M.E., Gonçalves A., Pinto C., Montalban X., Cavaco S. (2012). Olfactory dysfunction in multiple sclerosis: Association with secondary progression. Mult. Scler. J..

[8] Stewart M., Witsell D., Smith T., Weaver E., Yueh B., Hannley M. (2004). Development and validation of the Nasal Obstruction Symptom Evaluation (NOSE) scale. Otolaryngol. Head Neck Surg..

[9] Lachanas V., Tsiouvaka S., Tsea M., Hajiioannou J., Skoulakis C. (2014). Validation of the nasal obstruction symptom evaluation (NOSE) scale for Greek patients. Otolaryngol. Head Neck Surg..

[10] Hopkins C., Gillett S., Slack R., Lund V., Browne J. (2009). Psychometric validity of the 22-item Sinonasal Outcome Test. Clin. Otolaryngol..

[11] Lachanas V., Tsea M., Tsiouvaka S., Hajiioannou J., Skoulakis C., Bizakis J. (2014). The sino-nasal outcome test (SNOT)-22: Validation for Greek patients. Eur. Arch. Otorhinolaryngol..

[12] Frasnelli J., Hummel T. (2005). Olfactory dysfunction and daily life. Eur. Arch. Otorhinolaryngol..

[13] Simopoulos E., Katotomichelakis M., Gouveris H., Tripsianis G., Livaditis M., Danielides V. (2012). Olfaction-associated quality of life in chronic rhinosinusitis: Adaptation and validation of an olfaction-specific questionnaire. Laryngoscope.

[14] Kobal G., Hummel T., Sekinger B., Barz S., Roscher S., Wolf S. (1996). Sniffin’ Stick: Screening of olfactory performance. Rhinology.

[15] Konstantinidis I., Printza A., Genetzaki S., Mamali K., Kekes G., Constantinidis J. (2008). Cultural adaptation of an olfactory identification test: The Greek version of Sniffin’ Sticks. Rhinology.

[16] Oleszkiewicz A., Schriever V., Croy I., Hahner A., Hummel T. (2019). Updated Sniffin’ sticks normative data based on an extended sampleof 9139 subjects. Eur. Arch. Otorhinolaryngol..

[17] Langdon D., Amato M., Boringa J., Brochet B., Foley F., Fredrikson S., Hämäläinen P., Hartung H.-P., Krupp L., Penner I.K. (2012). Recommendations for a Brief International Cognitive Assessment for Multiple Sclerosis (BICAMS). Mult. Scler..

[18] Polychroniadou E., Bakirtzis C., Langdon D., Lagoudaki R., Kesidou E., Theotokis P., Tsalikakis D., Poulatsidou K., Kyriazis O., Boziki M. (2016). Validation of the Brief International Cognitive Assessment for Multiple Sclerosis (BICAMS) in Greek population with multiple sclerosis. Mult. Scler. Relat. Disord..

[19] Artemiadis A., Bakirtzis C., Chatzittofis A., Christodoulides C., Nikolaou G., Boziki M.K., Grigoriadis N. (2021). Brief international cognitive assessment for multiple sclerosis (BICAMS) cut-off scores for detecting cognitive impairment in multiple sclerosis. Mult. Scler. Relat. Disord..

[20] Smith A. (1982). Symbol Digit Modalities Test: Manual.

[21] Messinis L., Bakirtzis C., Kosmidis M.H., Economou A., Nasios G., Anyfantis E., Konitsiotis S., Ntoskou A., Peristeri E., Dardiotis E. (2021). Symbol Digit Modalities Test: Greek Normative Data for the Oral and Written Version and Discriminative Validity in Patients with Multiple Sclerosis. Arch. Clin. Neuropsychol..

[22] Vlahou C., Kosmidis M., Dardagani A., Tsotsi S., Giannakou M., Giazkoulidou A., Zervoudakis E., Pontikakis N. (2013). Development of the Greek Verbal Learning Test: Reliability, construct validity, and normative standards. Arch. Clin. Neuropsychol..

[23] Benedict R. (1997). Brief Visuospatial Memory Test-Revised: Professional Manual.

[24] Bakalidou D., Voumvourakis K., Tsourti Z., Papageorgiou E., Poulios A., Giannopoulos S. (2014). Validity and reliability of the Greek version of the Modified Fatigue Impact Scale in multiple sclerosis patients. Int. J. Rehabil. Res..

[25] Beck A., Steer R., Brown G., Antonio S. (2003). Manual for the Beck Depression Inventory-Fast Screen for Medical Patients.

[26] Hobart J., Lamping D., Fitzpatrick R., Riazi A.A.T. (2001). The Multiple Sclerosis Impact Scale (MSIS-29): A new patient-based outcome measure. Brain J. Neurol..

[27] Bakirtzis C., Nikolaidis I., Boziki M.-K., Artemiadis A., Andravizou A., Messinis L., Ioannidis P., Grigoriadis N. (2020). Cognitive Fatigability is Independent of Subjective Cognitive Fatigue and Mood in Multiple Sclerosis. Cogn. Behav. Neurol..

[28] Benedict R., Fishman I., McClellan M., Bakshi R., Weinstock-Guttman B. (2003). Validity of the Beck Depression Inventory-Fast Screen in multiple sclerosis. Mult. Scler..

[29] Mirmosayyeb O., Ebrahimi N., Barzegar M., Afshari-Safavi A., Bagherieh S., Shaygannejad V. (2022). Olfactory dysfunction in patients with multiple sclerosis; A systematic review and meta-analysis. PLoS ONE.

[30] Valsamidis K., Printza A., Constantinidis J., Triaridis S. (2020). The Impact of Olfactory Dysfunction on the Psychological Status and Quality of Life of Patients with Nasal Obstruction and Septal Deviation. Int. Arch. Otorhinolaryngol..

[31] Bsteh G., Steiger R., Tuovinen N., Hegen H., Berek K., Wurth S., Auer M., Di Pauli F., Gizewski E.R., Deisenhammer F. (2020). Impairment of odor discrimination and identification is associated with disability progression and gray matter atrophy of the olfactory system in MS. Mult. Scler..

[32] Valsamidis K., Printza A., Titelis K., Constantinidis J., Triaridis S. (2019). Olfaction and quality of life in patients with nasal septal deviation treated with septoplasty. Am. J. Otolaryngol..

[33] Printza A. (2022). Patient-Reported Outcome Measures in Diseases of the Head and Neck. J. Clin. Med..

[34] Printza A., Triaridis S. (2022). Is the ability of the Eating Assessment Tool (EAT-10) to screen for aspiration in patients with dysphagia depending on the patients’ disease?. Eur. Arch. Otorhinolaryngol..

[35] Benedict R.H.B., Amato M.P., DeLuca J., Geurts J.J.G. (2020). Cognitive impairment in multiple sclerosis: Clinical management, MRI, and therapeutic avenues. Lancet Neurol..

[36] Majde J.A. (2010). Neuroinflammation resulting from covert brain invasion by common viruses—A potential role in local and global neurodegeneration. Med. Hypotheses.

[37] Di Stadio A., Brenner M.J., De Luca P., Albanese M., D’Ascanio L., Ralli M., Roccamatisi D., Cingolani C., Vitelli F., Camaioni A. (2022). Olfactory Dysfunction, Headache, and Mental Clouding in Adults with Long-COVID-19: What Is the Link between Cognition and Olfaction? A Cross-Sectional Study. Brain Sci..

[38] Boziki M., Bakirtzis C., Giantzi V., Sintila S.-A., Kallivoulos S., Afrantou T., Nikolaidis I., Ioannidis P., Karapanayiotides T., Koutroulou I. (2021). Long-Term Efficacy Outcomes of Natalizumab vs. Fingolimod in Patients with Highly Active Relapsing-Remitting Multiple Sclerosis: Real-World Data from a Multiple Sclerosis Reference Center. Front. Neurol..

[39] Walsh M.C., Trentham-Dietz A., Newcomb P.A., Gangnon R., Palta M. (2012). Using propensity scores to reduce case-control selection bias. Epidemiology.

